# Supplementary material for: The RNA secondary structure of androgen receptor-FL and V7 transcripts reveals novel regulatory regions
Source: Nucleic Acids Res. 2024 Mar 30;52(11):6596–613. doi: 10.1093/nar/gkae220 (PMC11194067; doi:10.1093/nar/gkae220)
Supplement: gkae220_Supplemental_Files [file gkae220_supplemental_files.zip › Supplementary File S5.zip!/Supplementary File S5/FL_RScape.zip!/FL_RScape/AR_M58_1.R2R.sto.pdf]

AR_M58_1

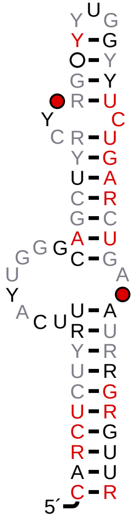

U
Y G
Y G
O Y
G Y
R U
Y
C
C R U
Y G
U A
C R
G C
A U
G G G C G
A
U
Y
A C U U A
R U
Y R
U R
C G
U R
C G
R U
A U
C R
5´
